# "Stealth dissemination" of macrophage-tumor cell fusions cultured from blood of patients with pancreatic ductal adenocarcinoma

**DOI:** 10.1371/journal.pone.0184451

**Published:** 2017-09-28

**Authors:** Gary A. Clawson, Gail L. Matters, Ping Xin, Christopher McGovern, Eric Wafula, Claude dePamphilis, Morgan Meckley, Joyce Wong, Luke Stewart, Christopher D’Jamoos, Naomi Altman, Yuka Imamura Kawasawa, Zhen Du, Loren Honaas, Thomas Abraham

**Affiliations:** 1 Gittlen Cancer Research Laboratories and the Department of Pathology, Hershey Medical Center (HMC), Pennsylvania State University (PSU), Hershey, PA, United States of America; 2 Department of Biochemistry & Molecular Biology, HMC, PSU, Hershey, PA, United States of America; 3 Department of Biology, Eberly College, University Park (UP), Pennsylvania State University, University Park, PA, United States of America; 4 Department of Surgery, HMC, PSU, Hershey, PA, United States of America; 5 Applications Support, Fluidigm Corporation, South San Francisco, CA, United States of America; 6 Department of Statistics, Eberly College, UP, PSU, University Park, PA, United States of America; 7 Department of Pharmacology and Biochemistry & Molecular Biology, Institute for Personalized Medicine, HMC, PSU, Hershey, PA, United States of America; 8 Department of Neural & Behavioral Sciences and Microscopy Imaging Facility, HMC, PSU, Hershey, PA, United States of America; University of Nebraska Medical Center, UNITED STATES

## Abstract

Here we describe isolation and characterization of macrophage-tumor cell fusions (MTFs) from the blood of pancreatic ductal adenocarcinoma (PDAC) patients. The MTFs were generally aneuploidy, and immunophenotypic characterizations showed that the MTFs express markers characteristic of PDAC and stem cells, as well as M2-polarized macrophages. Single cell RNASeq analyses showed that the MTFs express many transcripts implicated in cancer progression, LINE1 retrotransposons, and very high levels of several long non-coding transcripts involved in metastasis (such as MALAT1). When cultured MTFs were transplanted orthotopically into mouse pancreas, they grew as obvious well-differentiated islands of cells, but they also disseminated widely throughout multiple tissues in “stealth” fashion. They were found distributed throughout multiple organs at 4, 8, or 12 weeks after transplantation (including liver, spleen, lung), occurring as single cells or small groups of cells, without formation of obvious tumors or any apparent progression over the 4 to 12 week period. We suggest that MTFs form continually during PDAC development, and that they disseminate early in cancer progression, forming “niches” at distant sites for subsequent colonization by metastasis-initiating cells.

## Introduction

Pancreatic ductal adenocarcinoma (PDAC) is one of the most prevalent cancers worldwide, and is predicted to be the 2^nd^ leading cause of cancer deaths by 2030 [1]. PDAC is generally diagnosed at an advanced stage due to lack of early symptoms, precluding surgical excision, and there are no effective alternative treatments. As with most carcinomas, mortality is due to metastatic dissemination, and CTCs are observed in a high proportion of PDAC patients at all stages [2, 3]. While there are a number of models for what is termed the “metastatic cascade” [4], the nature of the CTCs which actually produce metastatic foci is not clear.

Perhaps the most widely accepted hypothesis underlying metastasis is that the primary tumor microenvironment (TME) induces an epithelial-to-mesenchymal transition (EMT) in a subset of epithelial cancer cells, that facilitates their escape into the bloodstream or lymphatics [5]. A number of studies for example have documented EMT-related changes (and loss of EpCAM expression) in CTCs [6–10]. In spite of recognized shortcomings [11, 12], CellSearch quantitation of numbers of EpCAM+ CTCs in peripheral blood has prognostic significance [13–15]. However, the picture remains incomplete: Which CTCs are the capable of initiating metastatic lesions (so called metastasis initiating cells, MICs), and how do MICs find suitable sites for growth of metastatic foci [5]? With regard to the former, a corollary idea is that the EMT-altered cancer cells at the periphery of a primary tumor facilitate liberation of cancer stem cells [5, 16, 17], which could represent the MICs. In this scenario, the overall number of CTCs would stochastically represent a much smaller subset of MICs. However, this story does not address the latter question: how MICs find suitable “niches” which allow them to establish metastases and proliferate [18].

An alternative theory for metastasis [19–22] involves fusion of macrophages with tumor cells (macrophage-tumor cell fusions, **MTF**s). With some sort of sorting, recombination, and/or reprogramming [23] of genetic material, this could produce neoplastic cells which have acquired the highly invasive phenotype of macrophages. There is considerable support for this notion from animal models, and some recent support from reports of human cancers [20], but how frequently this occurs is unknown and the basic premise seems to be at odds with the EMT/stem cell hypothesis [18].

We recently reported on MTFs cultured from blood from patients with early-stage and advanced melanomas [24]. The MTFs expressed multiple markers characteristic of M2-polarized macrophages, as well as epithelial, melanocytic and stem cell markers. When the melanoma MTFs were transplanted into mice as subcutaneous xenographs, they disseminated only to pancreas, where they formed what appeared to be benign islands of well-differentiated cells. Here we report on analogous MTFs cultured from blood of PDAC patients. These cells show expression of a similar combination of macrophage and epithelial/pancreatic/stem cell markers. Ultrastructural analyses revealed a macrophage-like morphology, with extensive autophagic vacuoles, etc. Single cell RNASeq analyses showed high levels of expression of various metastasis-related markers (particularly the MIF/CD44/CD74/CXCR4 signaling axis), as well as LINE-1 retrotransposons. In addition, the MTFs uniformly expressed very high levels of MALAT1, a long non-coding RNA transcript known to be involved in control of metastasis [25, 26], as well as additional long non-coding transcripts implicated in cancer progression. When the cultured PDAC MTFs were orthotopically transplanted into the pancreas in mice, they formed well-differentiated islands there. They did not form obvious tumors in any other distant locations. However, they were found to disseminate widely throughout multiple tissues, including liver, spleen, lung, submucosa, etc. They were found as single cells or small groups of cells and often appeared large and irregularly shaped. There was no apparent progression in number of cells in various tissues over the 4 to12 week period, although the only metastatic cells found in lung were observed at 12 weeks. The MTFs also appeared to alter their expression of some markers after dissemination.

## Results

### Immunophenotypic analysis of cultured MTFs

**Fig 1:**
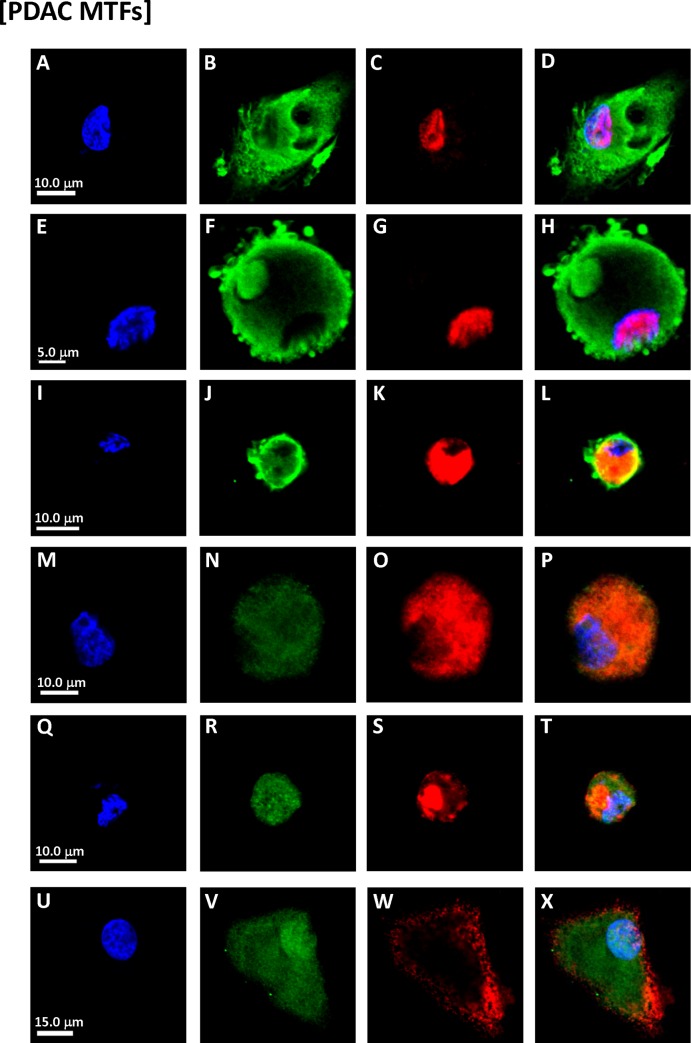
Immunostaining of cultured MTFs from PDAC patients. Representative confocal images of cultured MTFs from different PDAC patients. The cell populations showed uniform staining, and subcellular localization, of the various pairs of markers. Since the plates were sparsely populated, low power panoramic photomicrographs did not allow adequate visualization of the subcellular localizations, so photomicropraphs of individual cells were generally taken. Nuclei were stained with DAPI (Blue) shown in Panels [**A, E, I, M, Q** and **U**]. The same cells were also stained for various markers specific for pancreas, PDAC stem cell, or macrophage markers. Panels [**B, C, F** and **G**]: PDAC stem cell marker ALDH1A1 (Green) and pancreas marker ZG16B (red). Panels [**J, K, N** and **O**]: PDAC stem cell marker ALDH1A1 (Green) and pan-macrophage marker CD68 (Red). Panels [**R, S**]: Pancreas marker S100PBP (Green) and PDAC stem cell marker CD44 (Red). Panels [**V, W**]: M2-polarization macrophage marker CD206 (Green) and PDAC stem cell marker CD44 (Red). Composite images are shown in Panels [**D, H, L**, **P, T** and **X**].

**Fig 2:**
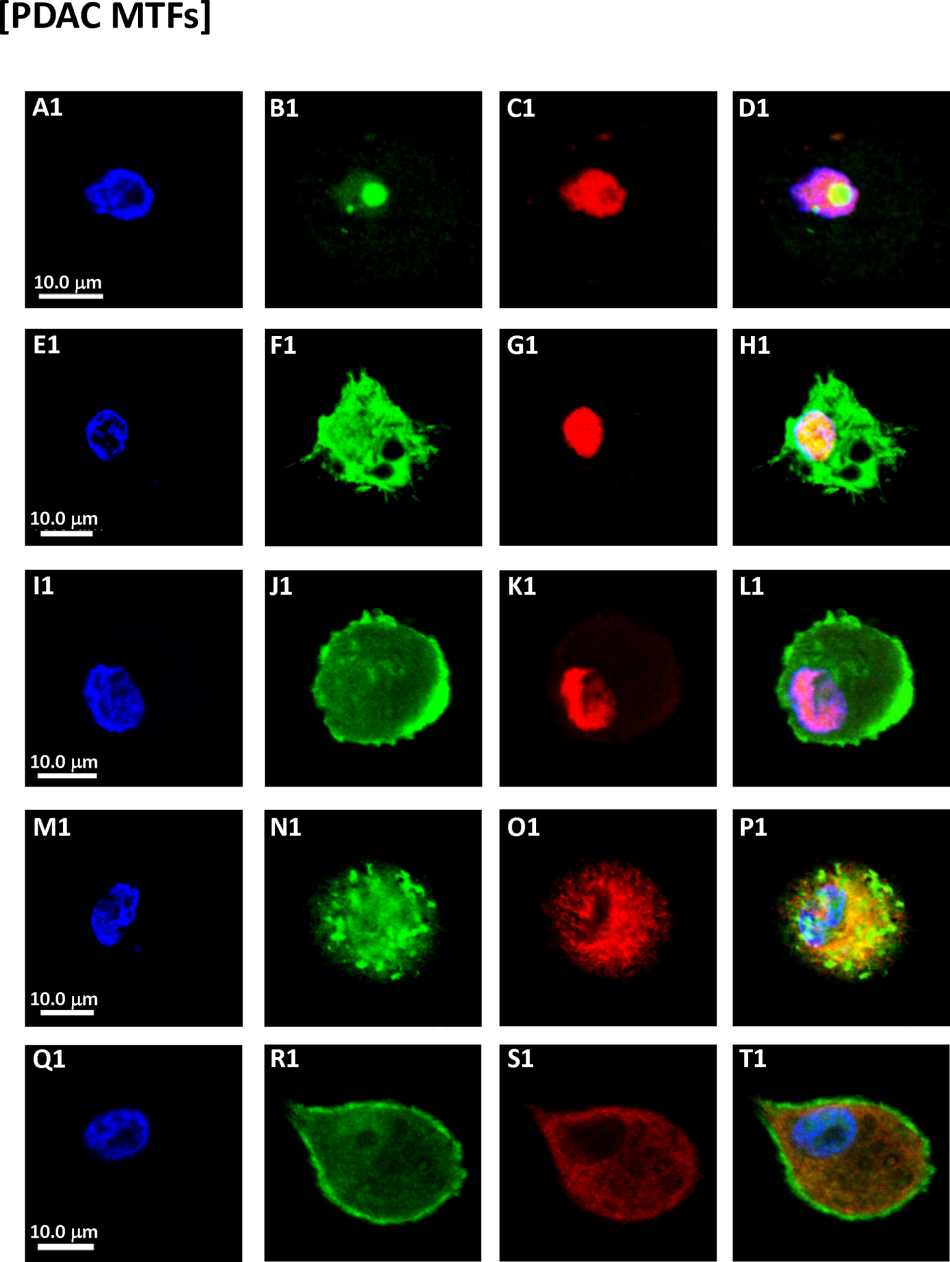
Immunostaining of cultured MTFs from PDAC patients. Representative confocal images of cultured MTFs from different PDAC patients. Nuclei were stained with DAPI (Blue) shown in Panels [**A1, E1, I1, M1** and **Q1**]. The same cells were also stained with various fluorescent markers specific for pancreas, macrophage, the pre-carcinogenic cytokine MIF and its receptor CXCR4. Panel [**B1, C1**]: MIF (Green) and pancreas-specific marker ZG16B (red). Nuclei had “tunnels” which were stained strongly for MIF. Panels [**F1, G1, J1** and **K1**]: CXCR4 (Green) and pancreas-specific marker ZG16B (red). Panels [**N1, O1, R1** and **S1**]: CXCR4 (Green) and M2-polarization macrophage marker CD204 (Red). Composite images are shown in Panels [**D1, H1, L1**, **P1** and **T1**].

Blood samples were obtained under an approved IRB protocol from patients with PDAC (some patients had early stage resectable disease, although most had advanced disease). Samples were processed as described, and cultured in standard media for ~4–6 weeks. Cells were quite sparse at the outset of culturing (perhaps a few cells/ml). Populations of cells grew from all preparations (~ 20), and they were fixed and stained for various pairs of markers and examined using confocal microscopy, including macrophage markers, pancreatic, epithelial, and pancreatic stem cell markers. The cell populations showed uniform expression (and localization) of the various pairs of human markers (**Figs 1 and 2**).

We also examined cultured MTFs for expression of the pro-carcinogenic cytokine MIF, because of MIF’ s prominent roles in M2 polarization of macrophages, the tumor microenvironment (TME), and cancer progression [27–31]. The cultured PDAC MTFs routinely stained positively for the MIF (Fig 2), with some very intriguing patterns of staining noted. Many of the individual nuclei appeared to have “tunnels” through them. These tunnels (invaginations) were lined by an intact nuclear envelope, and often contained cytoplasmic organelles including mitochondria, etc. (see below). The interior (cytoplasm) within these tunnels stained strongly for MIF, as determined using 3D confocal images (for example, see Panels A1 and B1 of Fig 2). Such tunnels had previously been observed in MTFs cultured from melanoma patient samples, as well as within melanomas in situ [24], and they are also evident in human PDACs (see below).

Given the robust immunostaining for MIF, we also examined the functionally related stem cell markers CXCR4 and CD44. CXCR4 is a non-cognate receptor for MIF [32, 33] and CD44 represents the signaling component of the MIF:CD74 receptor complex [34]. As with MIF, we observed strong expression of CXCR4 and CD44, indicative of pro-carcinogenic activities of the MIF/CD44/CD74/CXCR4 signaling pathway (Fig 2; see Discussion).

### Immunophenotypic analysis of primary human PDACs

**Fig 3:**
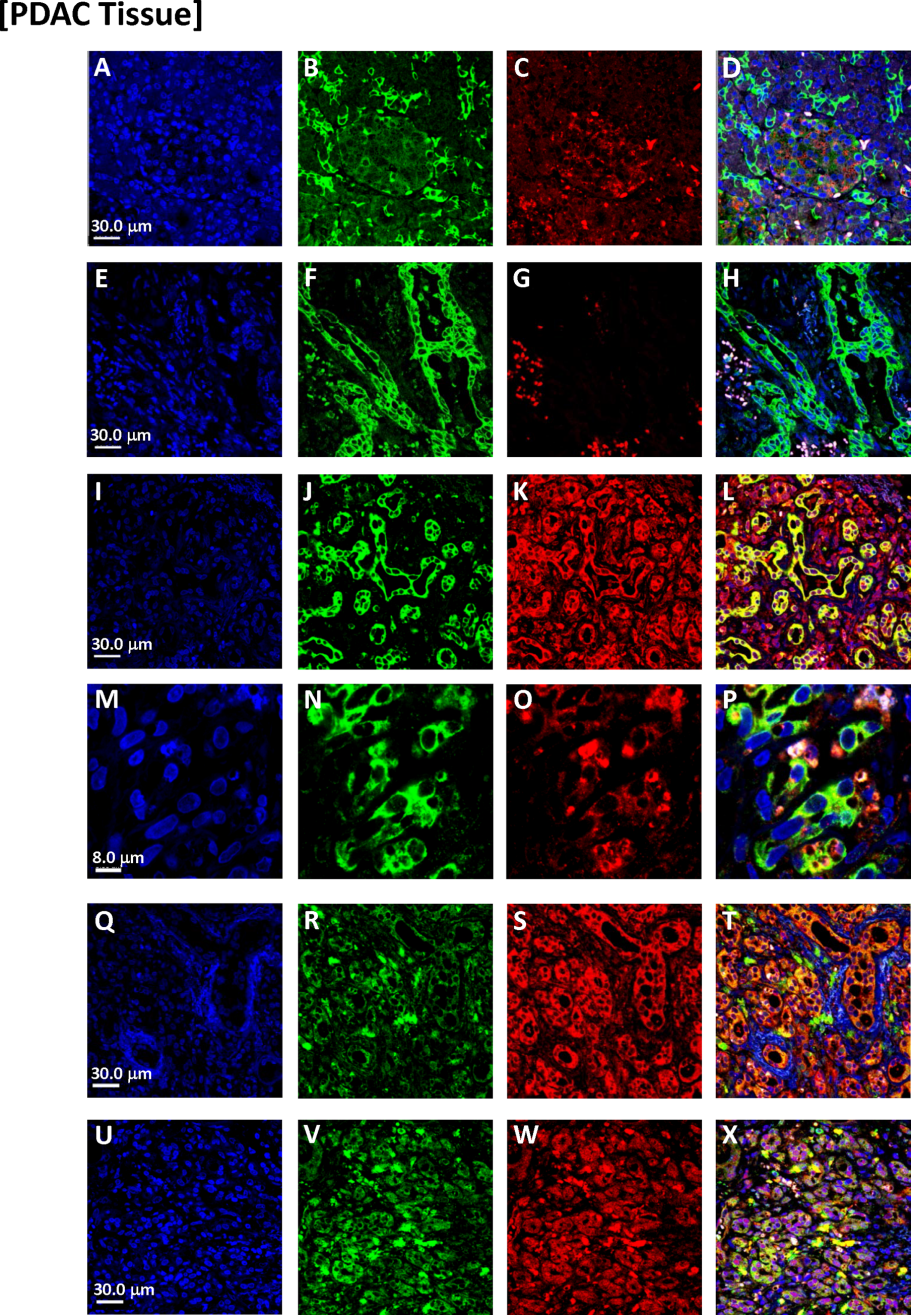
Representative confocal images of MTFs in PDAC tissues from different patients. Nuclei were stained with DAPI (Blue), shown in Panels [**A, E, I, M, Q** and **U**]. The same cells were also stained with various markers specific for pancreas, macrophage, or epithelial differentiation. Panels [**B, C, F** and **G**]: Epithelial marker pan-cytokeratin (Green) and M2-polarization macrophage marker CD206 (Red). Panels [**J, K**]: Epithelial marker pan-cytokeratin (Green) and M2-polarization macrophage marker CD163 (Red). Panels [**N, O**]: Epithelial marker pan-cytokeratin (Green) and M2-polarization macrophage marker CD204 (Red). Panels [**R, S**]: M2-polarization macrophage marker CD206 (Green) and epithelial marker EpCAM (Red). Panels [**V, W**]: M2-polarization macrophage marker CD206 (Green) and pancreas-specific marker ZG16B (Red). Composite images are shown in Panels [**D, H, L**, **P, T** and **X**].

**Fig 4:**
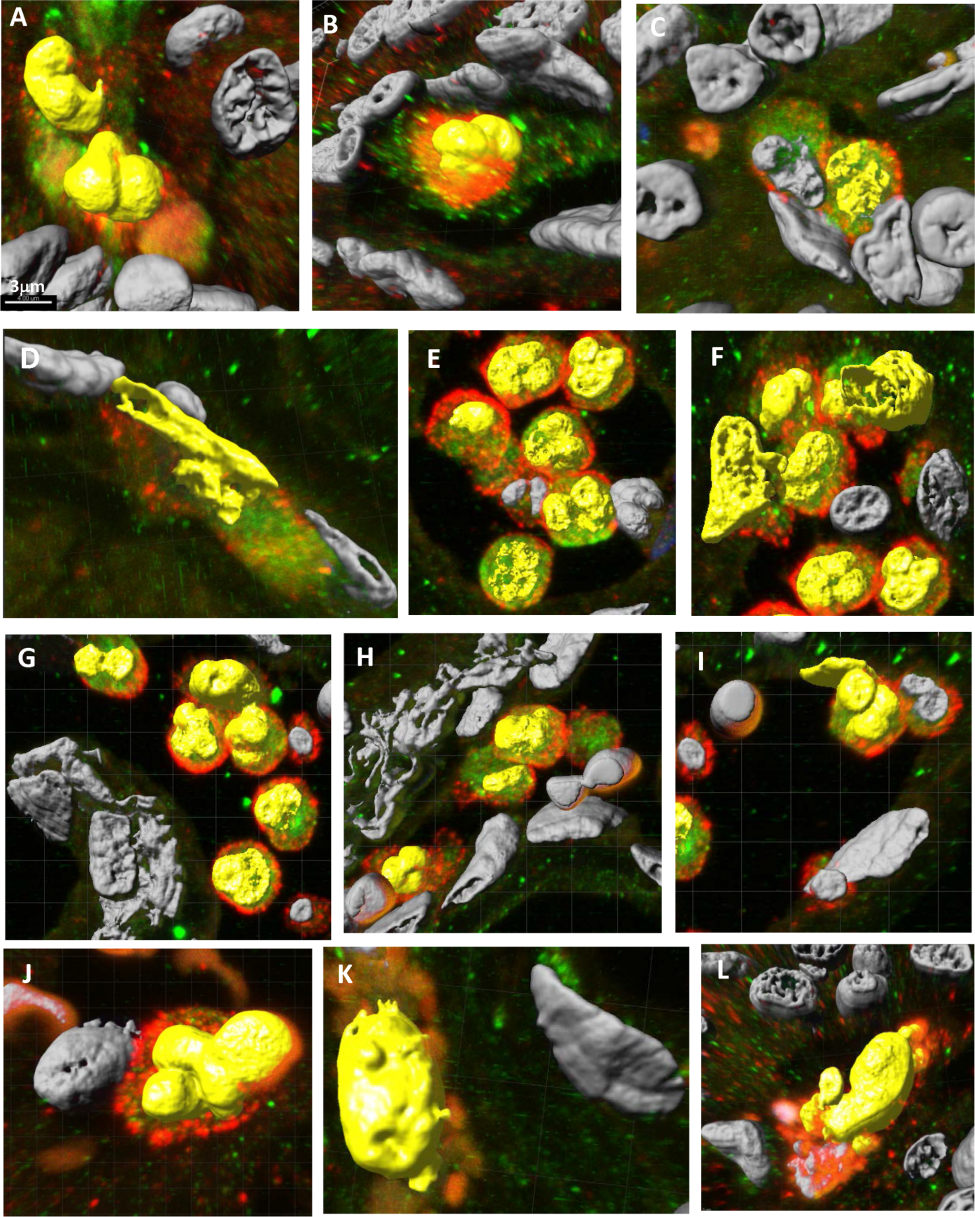
Representative 3D confocal images of MTFs in PDAC tissues from different patients which show various nuclear geometries (Yellow) of dual-stained cells. **[**Panels **A—I]:** Dual stained for pancreatic tumor marker ZG16B (Red) and macrophage marker CD 206 (Green)**; [**Panels **J & K]:** Dual stained for pancreatic tumor marker ZG16B (Red) and macrophage marker CD 204 (Green)**; [**Panel **L]:** Dual stained for pancreatic tumor marker S100BPB (Green) and macrophage marker CD 204 (Red).

**Fig 5:**
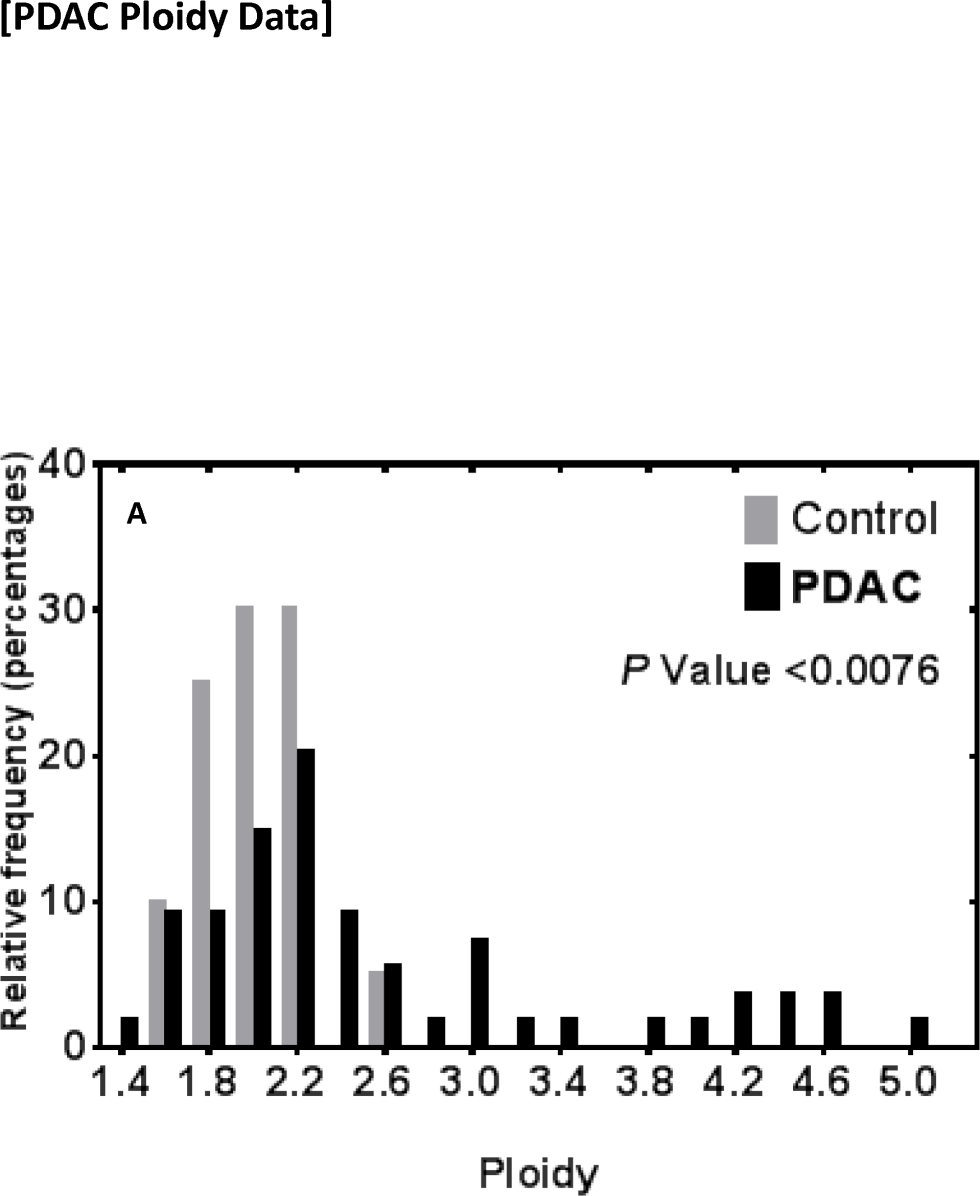
Ploidy analysis of dual-staining MTFs in PDACs. DNA content analysis of dual-staining cells was performed as described in PDACs in archival FFPE tissues. DNA content was also analyzed in adjacent “normal” pancreas (gray bars). Populations of MTFs from 2 patients, showed cells with DNA distribution peaks corresponding to “para-diploid” but with many aneuploidy cells distributed throughout the range, including some with DNA contents ranging up to 5n (black bars).

We observed analogous results in tissue specimens from primary human PDACs (**Fig 3**, Panels A-X). While there was morphologic heterogeneity in various regions of the PDACs, there was a surprisingly extensive subpopulation of cells that dually stained for various pairs of macrophage, pancreatic-epithelial, and stem cell markers (Fig 3). Invaginations (tunnels) were often evident in nuclei of the dual-staining cells (as was also apparent with DAPI-stained PDACs). With 3D confocal images, we also observed dual-staining of these cells for combinations of macrophage markers (CD204 or CD206) and pancreatic cancer markers ZG16B or S100PBP (**Fig 4**). We also conducted ploidy analysis of the apparent MTFs within PDACs in situ. We found that this population of dual-staining cells contained markedly abnormal DNA contents, with a large portion of the cells showing very irregular nuclei with aneuploid DNA content (**Fig 5**). These results are similar to those we previously described for melanoma-derived MTFs [24].

### Ultrastructural features of cultured MTFs

**Fig 6:**
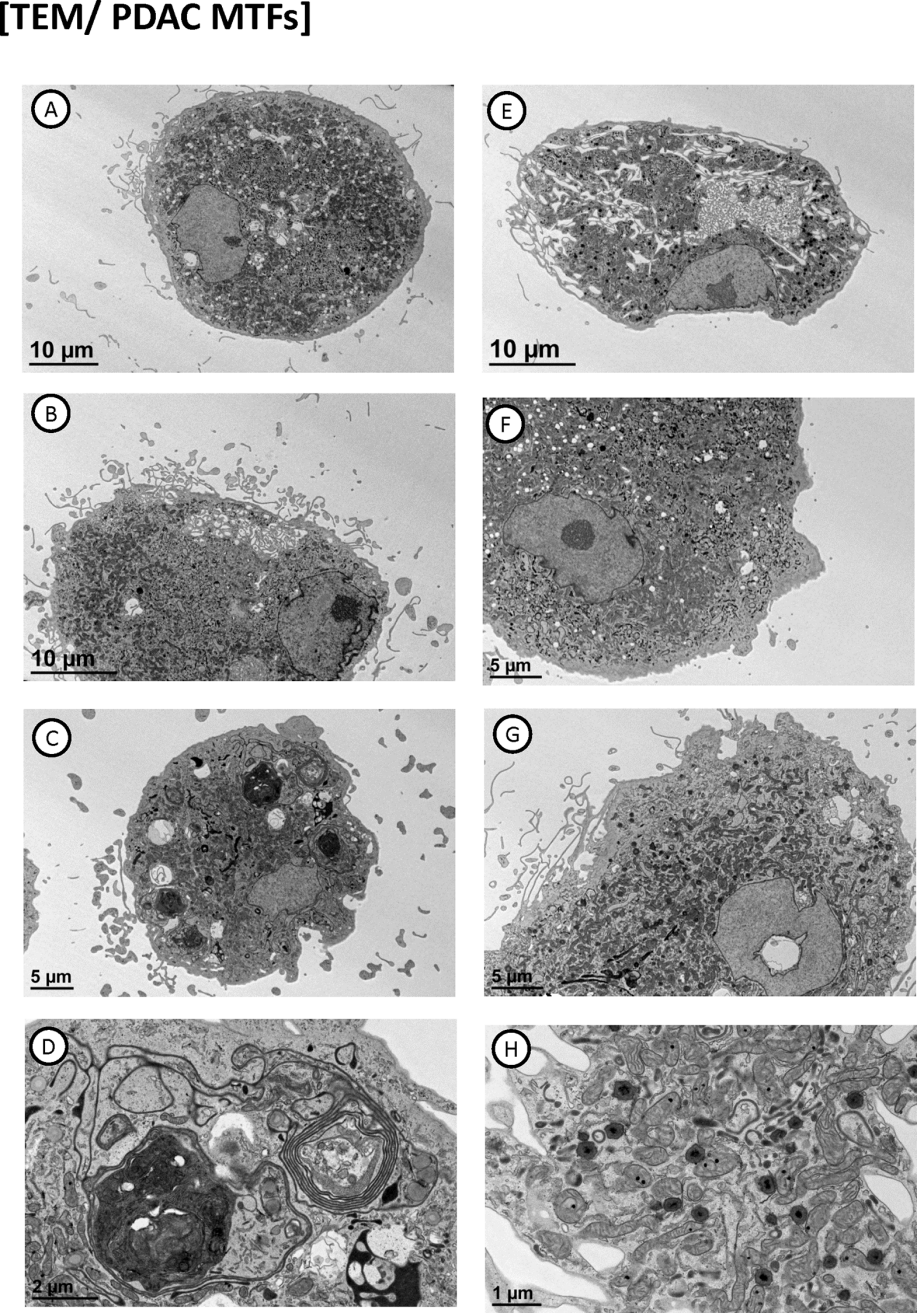
Transmission electron microscopy of cultured MTFs. Panels **A-H** show representative cells with distinctive features, including cytoplasmic “tunnels” through nuclei (Panel **G**). Focal hyper-dense regions of chromatin (Panel **A**, **E**, and **F**), autophagic bodies (Panel **D**), etc. are evident within the individual cells.

Ultrastructural examination of the MTFs via transmission electron microscopy (TEM) showed features characteristic of macrophages (**Fig 6**). The MTFs were generally large cells, which showed exuberant pseudopods, lamellipodia, and exocytosis. Nuclei generally showed very irregular (in fact, jagged) contours, as noted previously with melanoma MTFs [24]. Nuclei of the PDAC MTFs often showed “tunnels” through them (from ultrastructural examination, the concentrated staining for MIF is actually “extranuclear”, within the tunnels or invaginations of cytoplasm). MTFs contained large numbers of mitochondria, lysosomes, autophagic vacuoles, and various autolysomal breakdown products (including laminated bodies structurally comparable to lysosomes), and various structural remnants (Fig 6, Panels G & H). Heterogeneously-sized autophagic vacuoles containing chromatin (and micronuclei) were often a prominent feature, with some very dense remnant bodies (Fig 6H).

Notably, most nuclei also showed focal areas of condensed chromatin by TEM (Fig 6). They characteristically did not show fibrillar centers, or dense fibrillar or granular components generally seen in nucleoli. These regions may represent the ultrastructural correlate of focal areas of condensed “DAPI-intense” chromatin regions which have been reported in fusions between embryonic stem cells and somatic cells [35], and linked to malignancy in prostate cancer [36].

### Single cell RNASeq analyses of cultured MTFs

We also performed single cell RNA-Seq expression analyses on cells from 4 patients that both included and excluded assembled transcripts without coding potential (see Materials and Methods for details). Most of the transcripts identified were found in both analyses, although there were a few transcripts of interest which showed up differentially. These notably included CD74 (in the signaling pathway for MIF), which was one of the most highly expressed transcripts across all cells in all patients, as well as a few other genes such as HNRNPA2B1 (see below).

Expression clustering was done using complete linkage clustering with Euclidean distance across individual cells. The analysis that excluded transcripts without coding potential identified 5 clusters, which were common to all 4 patient datasets. Cluster 5 contained only 3 transcripts, all representing Long Interspersed Element-1 (LINE-1) retrotransposons (see below), which curiously encoded only ORF2. However, Cluster 1 also contained 14 distinct LINE1 transcripts, encoding both ORF1 and ORF2, as well as HNRNPA2B1, which is an HNRNP component which positively regulates LINE-1 retrotransposition (see Discussion). LINE-1 retrotransposons form the only autonomously active family of transposable elements [37]. Since the RNA transcripts contained both ORF1 and ORF2, this would appear to indicate that this family is actively engaged in moving DNA elements in these cells.

Cluster 4 contained only 3 transcripts, composed of ferritin light chain (FLT) and ferritin heavy chain (FTH1), which have been associated with several cancers [38–40]. Ferritin has been found in stroma and tumor-associated macrophages in breast cancer [41], where it was associated with increasing grade and stimulated proliferation of breast cancer cells via an iron-independent mechanism. FTH1 also serves as a novel marker for macrophages [42], and FTL is a prognostic marker in tumor-associated macrophages [43], along with CD163. In the analysis which included non-coding transcripts, FTL and FTH1 were present in cluster 2 (see **Table 1**).

**Table 1:** Cancer-related genes of interest.

Cluster 1		Cluster 2		Cluster 3	
Gene Name	Reference(s)	Gene Name	References	Gene Name	Reference(s)
PAG1	[132–134]	NANOG	[135]	TMSB10	[136–138]
ENO1	[139–141]	GPNMB	[142]	CTSD	[46]
CD38	[143–146]	ITGAX	[147]	CTSB	[47, 48]
EREG	[148, 149]	FTH1	[38–41, 43]	CTSS	[45]
DRAM1	[150–152]	FLT	[38–41, 43]	GPNMB	[142]
IREB2	[153, 154]	FUCA1	[155, 156]	S100A6	[157–160]
PEBP1	[161–164]	TMSB4X	[165, 166]	ANXA2	[167–169]
PTPN3	[170]	NABP1	[171, 172]	NBPF10	[173–175]
ARCN1	[176]	MALAT1 (lncRNA)	[56–59]	NEAT1 (lncRNA)	[54, 177–180]
NCOR1	[181]	CTSB	[47, 48]	HELLPAR (lncRNA)	[182]
SNRPD1	[183]				
DPYSL2	[184, 185]				
SRRM1	[186]				
NUPR1	[50–52]				
C16orf72	[187]				
HMGB1	[188]				
MGAT1	[189]				
HMGB1	[190–192]				
PARD6G	[193]				
ICK	[194, 195]				
ELK4	[196, 197]				
SIRPA	[198, 199]				
NUCKS1	[200–202]				
NUAK2	[203, 204]				
PYHIN1	[205]				
LAMP2	[206, 207]				
B4GALT5	[208–210]				
PRDX1	[211]				
TSPAN14	[212]				
CAP1	[213–216]				
DFFA	[217]				
HAUS2	[217, 218]				
MOB1A	[219]				
ITGB2	[220, 221]				
ST3GAL1	[222–224]				
ROCK1	[225–227]				
NIT2	[228]				
TFRC	[229]				
PTMA	[230]				
GPNMB	[142]				
CAPG	[231, 232]				
DDX51	[233]				
TFRC	[234]				
TRA2B	[235–237]				
SGK1	[238]				
S100A11	[239, 240]				
ACTR3B	[241]				
LSP1	[242–244]				
HERC4	[245]				
DOCK8	[246, 247]				
ANAPC15	[248]				
GINS4	[249, 250]				
SLC1A5	[251, 252]				
PTPN2	[253, 254]				
ADGRE2	[255, 256]				
GALNT6	[257, 258]				
MARCKS	[259–263]				
DIXDC1	[264]				
NBPF10	[173–175]				
TP53					
PHLDB1	[265, 266]				
PHACTR4	[267]				
ASAH1	[268–270]				
CCNI	[271, 272]				
**Long noncoding RNAs**					
NEAT1	[54, 177–180]				
LUCAT1	[273–275]				
MALAT1	[56–59]				
HELLPAR	[182]				
DDR1-AS1	[276, 277]				
KCTD21-AS1	[278]				

The analysis that included non-coding transcripts disclosed 3 clusters that were common to all 4 patient datasets, which we will discuss in more detail (the individual transcripts in the clusters are provided in Supplementary Material; as mentioned, most of the identified transcripts were found in both analyses).

Pathway enrichment analysis using WebGestalt identified a number of KEGG Pathways/disease categories among the highly expressed transcripts. These included Lysosome, Phagosome, Leukocyte transendothelial migration, regulation of actin cytoskeleton, antigen processing and presentation, NK cell-mediated toxicity, focal adhesion, B-cell receptor signaling, tight junction, osteoclast differentiation, pathways in cancer, bacterial invasion of epithelial cells, prostate cancer, and various infectious categories. The initial analysis identified immunologic diseases, stress, shock, lysosomal diseases, metabolic diseases and neoplasm metastasis, a category which contained 19 genes. However, a closer perusal of the data on a gene-by-gene basis identified a much larger number of genes which are implicated in cancer progression and metastasis (see Table 1). The largest cluster in the analysis that included transcripts without coding potential contained ~ 350 transcripts. It included a number of genes with obvious cancer relevance including CD44, catenin b1 (CTNNB1), vimentin (VIM), cathepsins B and S (CTSB, CTSS), MMP9, XIAP, JunD, numerous proto-oncogenes (REL, YES, SET, FGR, CRK), and HSP90AA1 (which is a chaperone which stabilizes MIF as a client). CD44 was uniformly expressed in all cells from all patients at very high levels; in fact, CD44 expression has been correlated with CD204 expression in PDAC, which serves as a predictor of survival [44].

When the individual genes were examined on a gene-by-gene basis, an additional 64 genes were identified which have been implicated in cancer progression and metastasis, as well as 6 long noncoding RNAs implicated in control of metastasis (see Table 1). For examples, Cathepsins B, D, and S were identified in cluster 3; they have a role in cancer progression and metastasis [45–48]. Nuclear protein 1 transcription regulator (NUPR1, also called candidate of metastasis 1) has been implicated in progression of pancreatic [49], bladder [50], liver [51] and non small cell lung cancers [52]. Nuclear paraspeckle assembly transcript 1 (NEAT-1) is a lncRNA that acts as a transcriptional regulator controlling expression of many genes implicated in progression and metastasis of many cancers [53, 54]. Other genes of interest included DNA damage-regulated autophagy modulator 1 (DRAM1), which plays a major role in regulating autophagic flux including fusion of lysosomes with autophagosomes and clearance of autophagosomes [55]. Also contained in the cluster were a number of macrophage markers (CD68, etc.).

In addition to these coding transcripts, the long noncoding transcript metastasis associated lung adenocarcinoma transcript 1 (MALAT1) was expressed in all cells from all patients. MALAT1 acts as a transcriptional regulator controlling expression of a number of genes involved in metastasis and prognosis in a variety of cancers [56, 57], including lung cancer [58] and in PDAC [59]. In fact, in terms of expression levels, MALAT1 and CD74 were the 6^th^ & 7^th^ most highly expressed transcripts (for reference, mitochondrial 16S rRNA was the most highly expressed transcript, followed by 4 other mitochondrial genes). In addition, all MTFs from one patient highly expressed a novel variant MALAT1 transcript (lacking ~ 300 nt from nt4600-4900, and apparently containing additional 5’ and 3’ sequences), which could have functional ramifications. Other identified lncRNAs included LUCAT1, which is important in non-small cell lung cancer, NEAT1, and HELLPAR. Two additional antisense RNAs were identified, with cancer relevant sense targets (Table 1).

### Dissemination of MTFs after orthotopic implantation into mice

**Fig 7:**
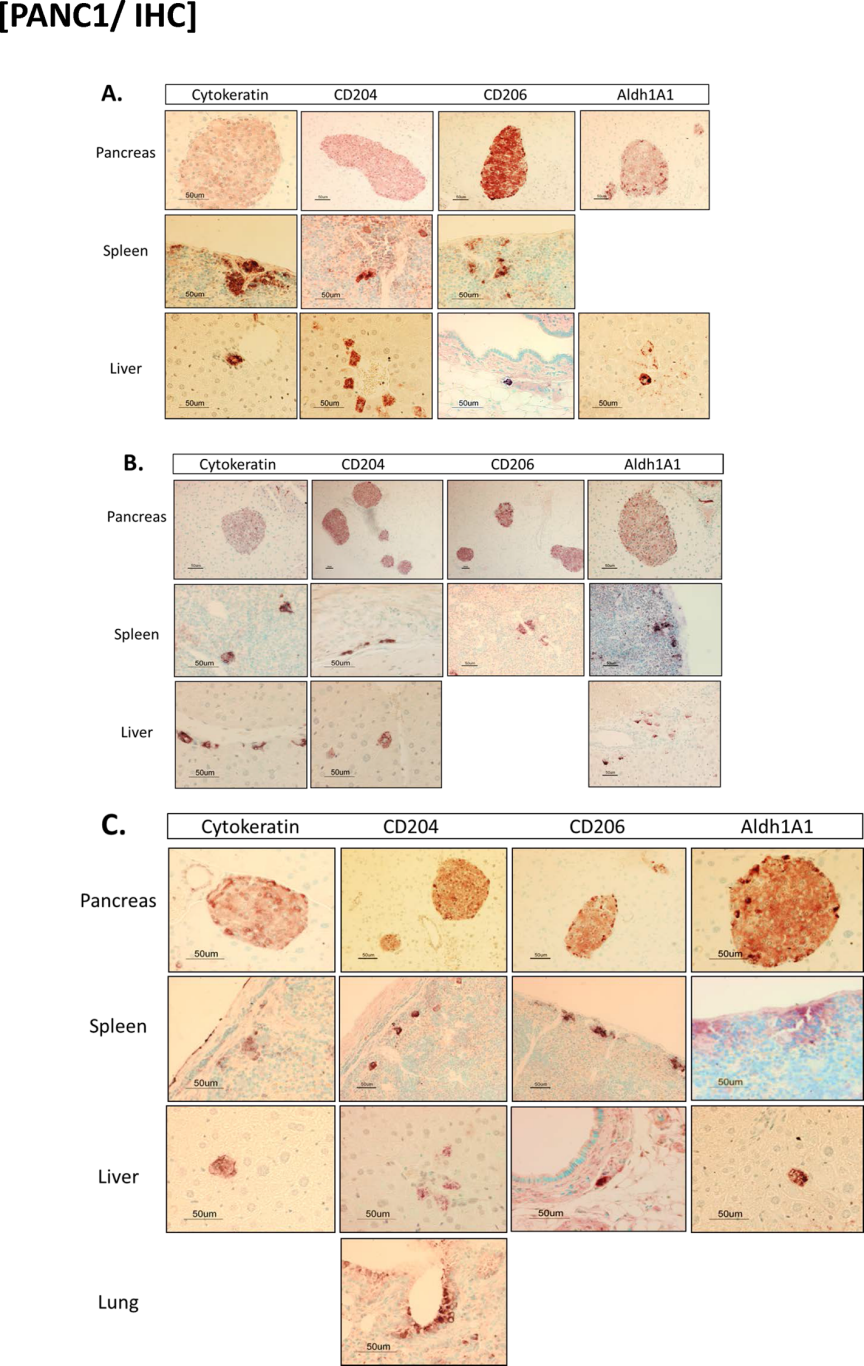
Immunohistochemical staining of mouse tissues after orthotopic transplantation of cultured MTFs. Human MTFs were cultured from blood as described, and orthoptically transplanted into mouse pancreas. After various periods of time, tissues were harvested and stained for an epithelial marker (cytokeratin, KRT), human macrophage M2 polarization markers CD204 and CD206, and human pancreatic stem cell marker ALDH1A1. Tissues (pancreas, spleen, and liver) are as indicated. Panel **A** shows samples harvested at 4 weeks after implantation, **B** shows samples after 8 weeks, and **C** shows samples after 12 weeks.

We then performed xenograft experiments, in which cultured MTFs were orthotopically transplanted into mouse pancreas, and mice were necropsied and examined 4, 8, or 12 weeks after transplantation (**Fig 7**). There was no evidence of overt tumors within the pancreata (or any other tissue sites) grossly in any of the animals. Microscopically, there were islands of well-differentiated MTFs within the pancreas, which appeared to be encased within lymphatic channels (Fig 7), at all time points examined. Although the cells were clearly of human origin (based on recognition of multiple markers by human-specific antibodies), they were much smaller and quite uniform in appearance in the pancreatic foci, and thus appeared quite different than when they were grown in culture (this was also true regarding melanoma MTFs). Heterogeneous staining for KRT, CD206, CD204, and ALDH1A1 was observed within the pancreatic foci, as was the case with the well-differentiated islands observed with melanoma cells after subcutaneous transplantation into nude mice [24].

In addition, however, we also observed individual cells (or small clusters of cells) which stained for KRT, or for human antigens ALDH1A1, CD204 and/or CD206 in various other tissues. In animals examined 4 weeks after transplantation, occasional individual cells, or small clusters of cells, showed expression of ALDH1A1 and CD204 in liver parenchyma and in spleen (Fig 7A), where staining was confined to small pericapsular clusters. Individual cells expressing CD204 were observed in focal areas within hepatic parenchyma; in general, their appearance was very similar to surrounding hepatocytes. Curiously, we did not observe staining for CD206 in hepatic parenchyma; however, we did observe individual cells expressing human CD206 in submucosal regions near bile ducts or vessels. Analogous results were also observed in animals sacrificed 8 weeks after transplantation (Fig 7B). At 12 weeks after transplantation, similar well-differentiated focal islands of cells were again observed within pancreas. Individual cells (or small clusters) expressing ALDH1A1 and CD204 were observed in liver and spleen, and occasional cells expressing CD204 were also observed in lung parenchyma (Fig 7C). Individual cells staining positively for CD204 and/or CD206 were also observed in submucosa in various locations (submucosa surrounding bile ducts, small bowel mucosa, etc.).

In general, cells observed in the various tissues were heterogeneous, and often appeared quite large and irregularly shaped. There did not appear to be any obvious progressive increase in numbers of cells over the 4 to 12 week period, although MTFs were only observed in lung after 12 weeks (Fig 7C).

**Fig 8:**
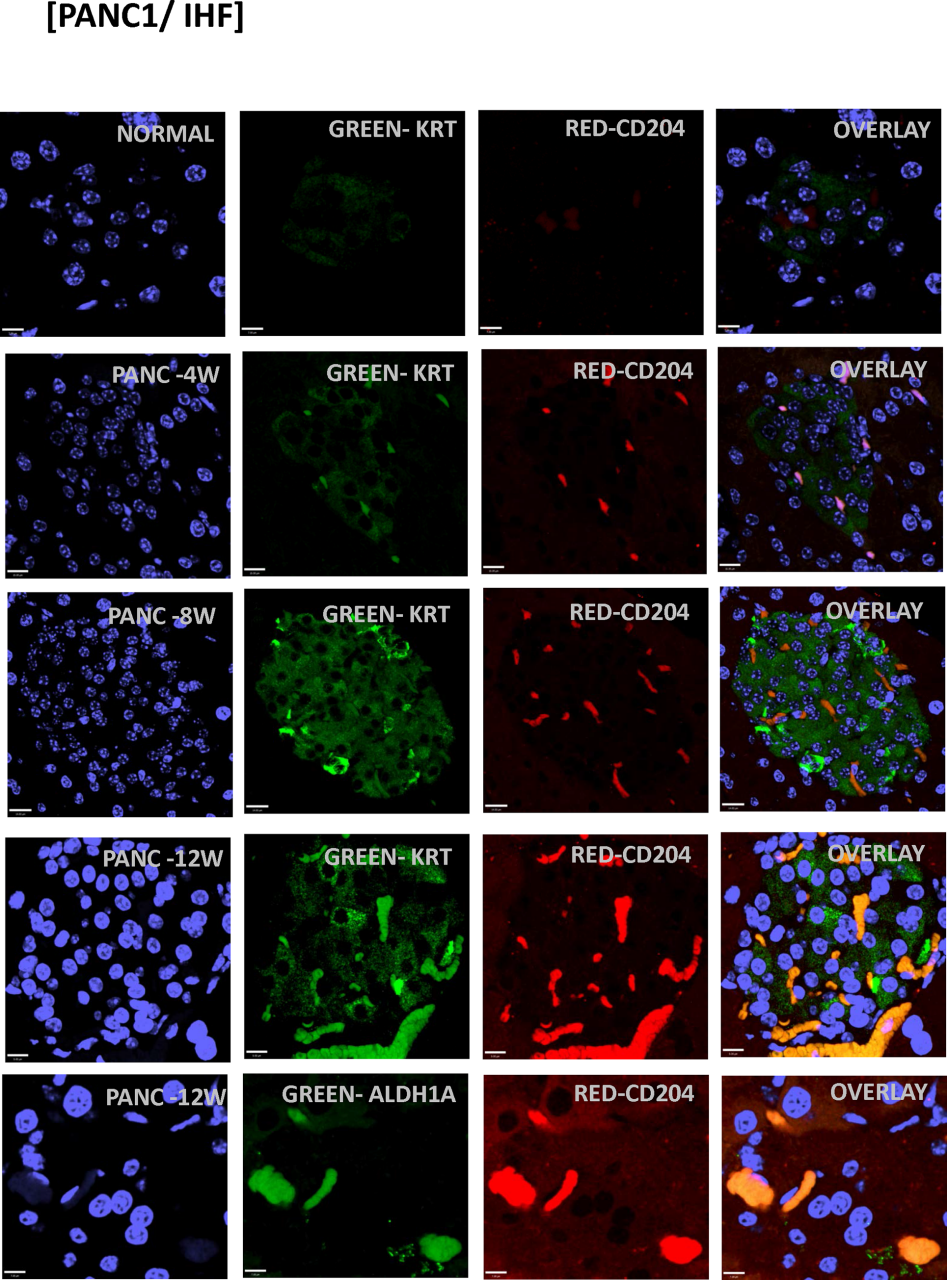
Immunostaining of human MTFs after orthotopic transplantation into mice. Cultured human MTFs were orthotopically transplanted into mouse pancreas and tissues were harvested at various times. Representative confocal images are shown from pancreas after 4, 8 and 12 weeks (**PANC-4W, PANC-8W, PANC-12W**) after orthotopic implantation. Nuclei were stained with DAPI (Blue). The same cells were also dual stained with various fluorescent markers specific for the epithelial marker cytokeratin (**KRT**), human macrophage M2 markers (**CD204/206**) or the human pancreatic stem cell marker (**ALDH1A1**).

**Fig 9:**
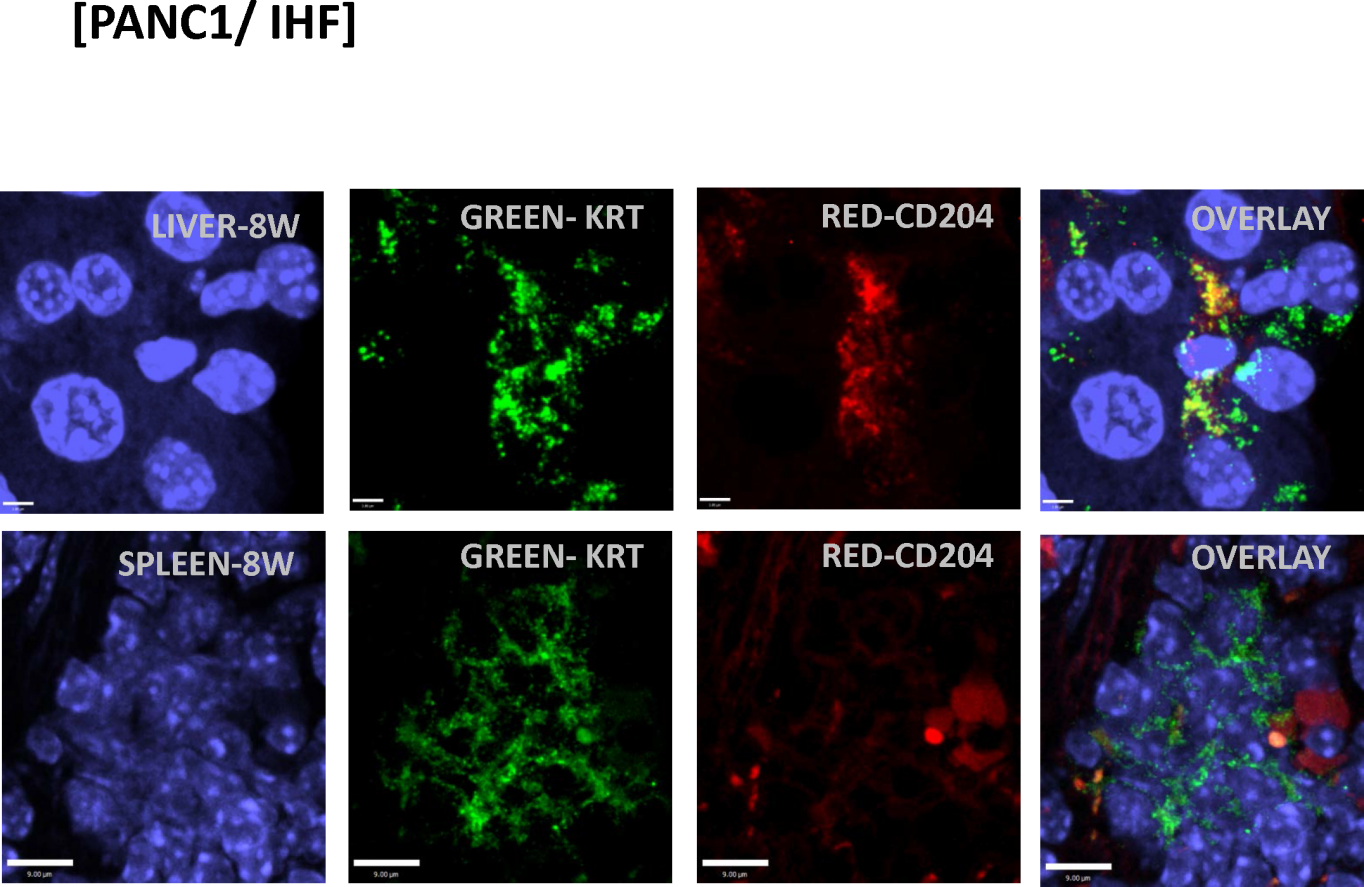
Immunostaining of human MTFs after orthotopic transplantation into mice. Cultured human MTFs were orthotopically transplanted into mouse pancreas. At 8 weeks following implantation, tissues were harvested and examined by confocal microscopy. Representative confocal images of liver or spleen (**LIVER-8W, SPLEEN-8W**) are shown. Nuclei were stained with DAPI (Blue). The same cells were also dual stained with various fluorescent markers specific for epithelial marker cytokeratin (**KRT**) or macrophage (**CD204/206**). Note that the CD204-expressing cell is no longer expressing KRT.

We observed analogous results with immunofluorescent confocal microscopy (**Figs 8 and 9**) for various markers, including KRT, CD206, CD204, CXCR4 and ALDH1A1 (Fig 8 shows pancreas from 8 week animals). Staining for additional human markers S100PBP, CXCR4, CD204, and CD206 identified obvious foci in pancreas of animals at 4, 8, and 12 weeks after implantation. We observed dual-staining scattered cells throughout other tissues, including spleen and liver (Fig 9). We also observed dual-staining for human pancreatic and macrophage markers in pancreas (Fig 9), including for S100BPB and ZG16B. No staining was observed in pancreas from control mice (Fig 8).

## Discussion

To briefly summarize some aspects of our results, cultured MTFs: 1) Uniformly co-express many epithelial/macrophage/pancreatic stem cell markers; 2) Show ultrastructural features suggestive of functional macrophages, with prominent autophagy; 3) Show high and consistent expression of the CD44:CD74:CXCR4:MIF signaling axis; 4) Show high, and apparently functional, expression of LINE-1 Retrotransposons (along with a positive regulator of their retrotranspositional activity) and the lncRNA MALAT1, based on RNASeq results; and 5) Were capable of “stealth” dissemination in vivo, whereby they metastasized to various distant sites and colonized the sites without forming apparent tumors.

The MTFs characteristically expressed a number of macrophage markers, many of which are characteristic of M2-polarized macrophages, such as CD163, CD204, and CD206. CD163 (Gene ID#9332) is a member of the scavenger receptor cysteine-rich superfamily, which may reflect proinflammatory cytokine production, and there are various reports linking its expression with poor prognosis in various cancers [60, 61]. CD204 (Gene ID#4481 or MSR1, the class A macrophage scavenger receptor type 1) is a functional receptor which mediates the endocytosis of low density lipoproteins, and its expression has also been linked with various cancers as well as with intralymphatic metastasis [62] CD206 (Gene ID#4360, also known as MRC1, the mannose receptor, C type 1) is involved in glycoprotein metabolism, and curiously has also been shown to be involved with CD44 in lymphatic trafficking [63]. We suggest that expression of these receptors may indicate use of alternative energy sources by transformed cells [64–69]. It is also of interest that while PANC-1 human PDAC cells strongly express CD204 and CD206, they do not express pan-macrophage markers like CD68 (Gene ID#968) or CD14 (Gene ID#929): CD68 and CD14 are unlikely to contribute to altered metabolic requirements.

The M2-polarization of cultured MTFs may have significant ramifications. M2-like macrophages are responsible for collagen degradation through a mannose receptor-mediated (CD206) pathway [70], and tumor associated macrophages (TAMs) generally acquire an M2-like phenotype that plays important roles in many aspects of tumor growth and progression [71–74], and M2-polarized TAMs have also been found to promote the EMT in various carcinomas [75, 76].

In fact, there are a growing number of reports of expression of macrophage markers on various types of cancer cells. For example, CD163 expression on rectal cancer cells is associated with early local recurrence and reduced survival time [77]. CD163 expression by breast cancer cells is related to early distant recurrence and reduced survival time [78]. In this regard, Shabo & Svanvik [79] reported that ~50% of breast cancer cells expressed CD163, and that a third of rectal cancer cells expressed it. CD163 was again associated with early distant recurrence in breast cancer, and with local recurrence in rectal cancer, and with reduced survival times in both. Expression of DAP12, a macrophage fusion receptor, was also associated with advanced tumor grade and higher rates of skeletal and liver metastasis, and overall shorter distant recurrence-free survival.

After orthotopic implantation in mice, the cultured MTFs were able to disseminate widely to various tissues, including (at least) pancreas, spleen, liver, and lung. They colonized the distant sites generally as single cells. After dissemination, some of the MTFs did not appear to continue to express all of the macrophage markers they expressed in primary cultures, and at times displayed some apparent plasticity in their morphology. For example, discordant expression of the CD204 and CD206 markers was observed in liver. Staining for CD204 was positive within hepatic parenchyma for individual cells and focal nests of cells in liver of animals at 4,8, and 12 weeks after transplantation (Fig 7). These positively staining cells were observed only in select regions of the livers, and they appeared as well-differentiated cells morphologically similar to hepatocytes. However, staining for CD206 was not observed in hepatic parenchyma at any of the time points. Scattered cells staining positively for CD206 were observed in submucosa of vessels and bile ducts (see Fig 8A and 8C); these cells were large, irregularly shaped cells with morphology more consistent with macrophages, which was also evident in confocal micrographs in pancreatic foci (Fig 8).

Ding et al. [80] reported that MTFs may acquire cancer stem cell properties in breast cancer cells. They noted expression of CD163 in breast cancer tissues, where it varied significantly and CD163 expression correlated with ER expression. These investigators created fusions between M2-polarized macrophages and breast cancer cell lines. The MTFs gained a CD44^**+**^CD24^**-/low**^ phenotype, overexpressed EMT related genes, and showed increased migration, invasion, and tumorigenicity (but reduced proliferation). There was some indication that the fusions were also associated with increased metastasis, although an increase in metastases was also observed with mixtures of the M2-differentiated U937D cells and breast cancer cell lines (both MCF7 and MDA-MB-23) without fusion, so the role or occurrence of fusion per se was not clear. The cultured MTFs described here appear to mirror these properties.

### MIF in the TME and cancer progression

We also observed activation of the MIF signaling axis in the cultured PDAC MTFs. MIF has important roles in the TME as well as progression of many cancers. MIF levels are associated with an increased incidence of a large number of cancer types [28, 32, 81–86]. MIF serves as a non-cognate ligand for CXCR4 [32, 33]: MIF (and CXCR4) in the TME are adverse prognostic indicators [28], and it can induce CXCR ligand and regulators of macrophage infiltration, especially CD44 [87]. In PDAC, MIF has been shown to induce the EMT [88, 89], enhance tumor aggressiveness, and predict clinical outcome in resected PDACs [27]. M2 polarized TAMs also have prognostic importance in PDAC [73, 90], and targeting TAMs decreases tumor-initiating cells, relieves immunosuppression, and improves chemotherapeutic responses [91]. MIF controls alternative activation of TAMs to M2-polarization [88]; In turn, co-culturing M2-polarized TAMs with PDAC cells strongly induces the EMT [89].

MIF expression is up-regulated during hypoxia via an HRE found in the 5’-UTR of the gene [92, 93]. Proteomic and tissue array profiling has identified elevated hypoxia-regulated proteins in microdissected PDAC nests vs. normal ducts [94], prominently including MIF, which showed excellent ROC curves in discriminating PDACs from non-malignant lesions. MIF is a direct transcriptional target of HIF1a, and loss of MIF results in inefficient HIF1a stabilization induced by hypoxia [95]. In this context, CSN5 of the COP9 signalosome interacts with MIF in PDAC cells to stabilize HIF1a. Intracellularly, MIF is also stabilized by complexing with HSP90 chaperone [83, 86], which was found in cluster 2 in the RNASeq data analysis (Cluster 2 also contained CD74). Cancer cells contain constitutive endogenous MIF-HSP90 complexes, and inhibition of HSP90 function results in apoptosis, which can be overridden by ectopic MIF expression. In fact, the metastasis-promoting CD44 in PDACs is actually the signaling component of the MIF-CD74 receptor complex [34]. MIF signaling through CD74 promotes sustained ERK activation, which corresponds to the main outcome from Ras mutations, mutations which figure so prominently in PDAC (see [96]). Inhibition of MIF using siRNAs leads to apoptosis in PDAC cells [97]. Long et al. [98] developed a unique mouse model for PDAC lymphatic metastasis. They developed a subline from the BxPC-3 PDAC cell line via serial passages in nude mice. The subline showed increased migration, invasion, and invasive ultrastructural characteristics. Metastasis-related gene alterations found in the subline were very limited but included up-regulation of MIF. We speculate that MIF expression in distant tissues by disseminated MTFs may prepare “niches” for subsequent colonization by metastasis-initiating cells.

### CTCs in PDAC

CTC isolation and analysis has become an active area of translational cancer research [99]. However, unlike breast, prostate and lung cancers, comparatively little is known about CTCs in pancreatic cancer, although some literature is appearing [2, 3, 100]. Recent literature using a novel KPCY mouse strain (KPC mice with the addition of a *Rosa*^*YFP*^ allele) demonstrated that PDAC cells enter the bloodstream unexpectedly early, during the pre-cancerous PanIN lesion stage and before carcinoma was evident. The circulating, pancreas-derived cells exhibited a mesenchymal phenotype (although somewhat different from that described by Ting et al. [100]), and the number of circulating cells was increased in the presence of chronic pancreatitis [101]. The murine study was recently followed up by a prospective human study that captured circulating cells of pancreas-derived lineage from the blood of patients with pre-cancerous pancreatic lesions but no clinically detectable cancer. Since pancreas-derived cells were present in the blood even at pre-cancerous stages, the authors suggested that these cells could initiate metastatic lesions [102], although many aspects remain unclear. When patient populations with no cancer, pre-cancerous lesions or confirmed carcinoma were compared, the number of circulating, pancreas-derived cells significantly increased with disease progression. These results, as well as other studies [2], provide evidence that cells of pancreatic origin are released into the bloodstream at significantly earlier stages of tumor formation than previously thought. We point out that these cells could well include MTFs.

### Ploidy in PDAC

Here we found that apparent MTFs were a prominent component in human PDACs, and that they specifically show a wide range of aneuploid DNA content (Fig 5), with a large proportion of the cells containing greater than 3n. DNA index is known to be a strong prognostic factor in PDAC patients [103, 104] where 50–75% of patients showed non-diploid DNA contents. There is also a clear relationship between DNA content and survival in PDAC patients. Lymph node involvement was seen in 36% of patients with diploid tumors, vs. 79% of those with aneuploid tumors. 32% of patients with a diploid tumor survived at least 1 year, whereas none of the patients with aneuploidy tumors did [105], and aneuploidy showed a significant association with decreased cumulative survival. Tsavaris et al. [106] found that PDAC patients with ploidy score greater than 3.6 had 5X higher probability of death within a given time-frame compared with patients with ploidy score of less than 2.2, and those with an intermediate ploidy score (2.2–3.6) had a 6.3X higher probability of death compared with patients with ploidy score less than 2.2. A similar relationship was found for patients with late stage colorectal cancers [107]. Given that apparent MTFs appear to constitute a considerable portion of PDAC populations, these relationships may reflect higher MTF proportions.

### DNA reconciliation and mobile elements in MTFs

When fusion of 2 different cell types occurs, two distinct cellular programs need to be merged. This process has been referred to as symphiliosis, the process of intracellular reconciliation [108], which suddenly produces new clones with emergent phenotypes. Cancer cells can transduce adjacent TME cells in vivo, and it has been suggested that in vivo fusion discloses genes implicated in tumor progression [109]. Here, we note that the MTFs present a surprisingly uniform immunophenotypic profile, in spite of the often huge differences in DNA content. They appear to be undergoing cellular reconciliation, with prominent autophagy including nucleophagy [110] within autophagosomes which have sequestered chromatin and even micronuclei. Many aspects of micronuclei formation have been detailed [111], and degradation of micronuclei via autophagosomes has previously been reported [112], where it was speculated that removal of micronuclei may contribute to the genome-stabilizing effects of autophagy. Nucleophagy has been reported in various laminopathies [113] and seems to be beneficial for cell survival [113, 114]. The defacto depolyploidization process seems to involve macroautophagy-aided elimination of chromatin, which somehow includes sorting out what will be eliminated.

An additional finding of significance was the uniform expression of various LINE-1 retrotransposons, as well as a positive regulator (HNRNPA2B1) of their expression. Cluster analysis disclosed a considerable number of Long Interspersed Element-1 (LINE-1), some of which included both ORF1 and ORF2. LINE-1 retrotransposons form the only autonomously active family of transposable elements [37], and specific classes of LINE-1 elements are up-regulated during reprogramming and in transformed cells [115]. Since the RNA transcripts contained both ORF1 and ORF2, this would appear to indicate that this family is actively engaged in moving DNA elements in these cells, which presumably comprises part of the “reconciliation” of DNA. Activation of LINE-1 retrotransposons has also been linked to metastasis and the EMT [116].

We note here that most (but not all) of the patient samples analyzed here were relatively late stage PDACs. Although literature clearly suggests that CTCs (and/or MTFs) should be present in the circulation in early stage disease, this needs to be established before potential therapies targeting circulating MTFs could be deployed to block metastatic spread. We are currently working on PDAC MTF-targeted nanoliposomal delivery of an HSP90A inhibitor, with the goal of eliminating MIF and its signaling axis.

## Materials and methods

This Human Subjects Research was approved by the Pennsylvania State University/Hershey Medical Center IRB, and informed written consent was obtained from all participants. Peripheral blood (10–15 ml) was obtained from ~20 patients with pancreatic ductal adenocarcinoma (plus healthy volunteers). Our patient population is ~ 10% Hispanic or Latino, with ~ 10% Black or African American, with a targeted enrollment of 50% male/female.

### Isolation and culturing of MTFs

An initial CTC enrichment was performed using Oncoquick porous membrane gradients as previously described [24, 117], or using Ficoll-Paque PLUS gradients (GE Healthcare). The CTC-enriched fractions were rinsed in PBS, and then plated onto standard culture dishes and cultured in RPMI 1640 + 10% bovine serum. After 24 hours, plates were carefully rinsed to remove non-adherent cells, and new medium was added and cultures were continued for various periods of time. The cells appeared large and “epithelioid” at 24 h, and retained the same basic morphology throughout culturing; this was also noted in a previous publication which also examined CTCs captured on filters [117]. Cultures were generally grown for 4–6 weeks, initially in 25 cm plates and later in 75 cm plates. No cells grew in cultures of peripheral blood obtained from normal volunteers.

### Immunofluorescent staining of MTF cultures and PDACs

After various culture times, cells were transferred to chamber slides, and after growth for 24 h Immunochemical staining was performed for a variety of different markers (see Table 2).

**Table 2:** Antibodies used for immunophenotypic characterizations.

Antigen	Company	Source	Specificiy
ALDH1A1	Santa Cruz Sc22588	Goat polyclonal	human
CD44	R & D Systems Mab7045	Mouse monoclonal	human
CD68	Abcam Ab955	Mouse monoclonal	human
CD163	Abcam Ab87099	Mouse monoclonal	human
CD163	AbD SeroTec MCA1853T	Mouse monoclonal	human
CD204/MSR1	Sino Biological 10427	Mouse monoclonal	human
CD206/MRC1	AbD Serotec MCA2155T	Mouse monoclonal	human
CXCR4	Abcam AB181027	Rabbit polyclonal	human/mouse
CXCR4	Abcam Ab1670	Goat polyclonal	human/mouse
EpCAM	EMD Millipore OP-187	Mouse monoclonal	human
EpCAM	Cell Signaling Tech	Mouse monoclonal	human
MIF	Santa Cruz Sc-20121	Rabbit polyclonal	human/mouse
S100PBP	Atlas Antibodies HPA027328	Rabbit polyclonal	human
Pan-KRT	Santa Cruz Sc-15367	Rabbit polyclonal	human/mouse
ZG16B	R & D Systems Mab7777	Mouse monoclonal	human

Fluorescently-labeled secondary antibodies used for the different primary antibodies were purchased from Enco Scientific Services Limited: They included (as necessary):

115-545-062, Alexa Fluor 488-AffiniPure Goat Anti-Mouse IgG; 115-585-062, Alexa Fluor 594-AffiniPure Goat Anti-Mouse IgG; 305-545-003, Alexa Fluor 488-AffiniPure Rabbit Anti-Goat IgG; 305-585-003, Alexa Fluor 594-AffiniPure Rabbit Anti-Goat IgG; 711-545-152, Alexa Fluor 488-AffiniPure Donkey Anti-Rabbit IgG; or 711-585-152, Alexa Fluor 594-AffiniPure Donkey Anti-Rabbit IgG.

Generally, cells were stained for various combinations of 2 markers, as well as DAPI, and examined by confocal microscopy. PANC-1 (ATCC, CRL-1469) cells were also examined in parallel. Controls included no primary antibody as well as normal tissues.

For cultured MTFs, cells were grown overnight in 8-well coated chamber slides (Lab-Tek II CC^2^) and immunostaining was performed as previously described [24, 117]. Blocking was performed in PBS + 1:50 dilution of serum (from the species the secondary antibody was produced in) for 1 h at room temperature (RT). 200 microliters/chamber well of primary antibody solutions (1:200 dilution) was used. The balance of the staining protocol was performed in the dark, or with slides protected from light. 200 microliters of secondary antibody (1:500 dilution in blocking solution) was used. To counterstain nuclei, 200 microliters of DAPI solution (1:30,000 in PBS) was added to each well, and incubated for 5 min at RT in the dark. Cells were again washed 3X for 3 min each in PBST, and then a final rinse for 10 min at RT with PBST, and the final wash was again removed by inversion wicking. Coverslips were mounted using 3 drops of ProLong Gold Antifade mounting medium (Invitrogen), pre-warmed to RT, and slides were stored in the dark at 4 C. The mounting medium minimizes the refractive index mismatch of the lens immersion liquid (Cargile oil, refractive index of 1.52).

In addition, slides from formalin-fixed paraffin embedded (FFPE) primary human PDAC tumors were also stained for the various markers as described.

### Subcutaneous implantation of cultured human MTFs into athymic mice

All animal studies were performed under protocols approved by the PSU IACUC.

Male, 6-week old athymic (nu/nu) mice were purchased from Charles River. For surgical implantation of human PDAC CTCs, animals were fully anesthetized with Ketamine-Xylazine (120 mg/kg—4 mg/kg, i.p.), an incision was made on the left flank and the pancreas was exteriorized. Cultured CTCs from individual PDAC patients, 1.5 x 10^5^ cells in 50 microliters of HBSS (n = 3 mice / patient sample) were injected into the pancreas. The incision was closed with surgical wound clips and the animals were monitored for any ill effects. Experiments used cultured MTFs from a number of separate PDAC patient samples, which had been grown in culture for about 4 weeks. Mice were monitored for any outward sign of tumor formation, and they appeared normal throughout. At 4, 8, or 12 weeks after transplantation, mice were fully anesthetized with Ketamine–Xylazine and euthanized by CO_2_ administration and cervical dislocation. Lungs, liver, spleen, and pancreas were excised and fixed in 10% neutral buffered formalin for 24 hrs. After equilibration in 70% ethanol, tissues were embedded in paraffin and sectioned. Hematoxylin/eosin staining and immunohistochemical staining for various human macrophage, pancreatic and epithelial cell markers was done on serial tissue sections.

Tissue specimens from xenografted mice were also stained for the macrophage, stem cell, and epithelial markers (and DAPI) described above, generally using antibodies specific for the human antigens, and examined via IHC and/or confocal microscopy. These included sections from FFPE blocks of various tissues from the mice (liver, lung, pancreas, spleen). In addition, immunohistochemistry was also performed on the various tissues, using multiple antibodies which were specific for the human antigens. For these studies, secondary antibodies and reagents for immunoperoxidase staining were purchased from Vector Laboratories (ImmPRESS Anti-rabbit Ig (peroxidase) MP-7401, and ImmPRESS Anti-mouse Ig (peroxidase) MP-7402). Peroxidase substrate studies used ImmPACT DAB Substrate Kits (SK-4105). Controls included staining of normal mouse tissues as well as no primary antibody.

### 3D confocal microscopy, image acquisition, and image processing

Confocal images of fluorescently labeled cells were acquired with a Leica AOBS SP8 laser scanning confocal microscope (Leica, Heidelberg, Germany) using a high-resolution Leica 40X/1.3 Plan-Apochromat oil immersion objective. The laser lines used for excitation were continuous wave 405 (for DAPI), 80 MHz pulsed 499 nm (for Alexa 488), 80 MHz pulsed 591 (for Alexa 594). These laser lines were produced by UV diode, 80 MHz white light laser (Leica AOBS SP8 module) respectively and the respective emission signals were collected sequentially using AOBS tunable filters as follow; 410–480 nm for DAPI (this exclude possible RNA bound DAPI emission which occurs above 500 nm), 504–571 nm for Alexa 488, 597–751 nm for Alexa 594 and 650–790 nm for TO-PRO-3. All images, spectral data and DNA ploidy measurement data were generated using the highly sensitive HyD detectors (with time gated option) in descanned mode and the photon counting mode was particularly used for collecting signals from DAPI for DNA ploidy measurements. The backscattered emission signals from the sample were delivered through the AOBS tunable filter (to remove irradiated laser), the detection pinhole set to 1 Airy unit (to obtain optimal lateral and axial resolutions), spectral dispersion prism, and finally to the HyD detectors. The width of the slits in front of each HyD could be software adjusted so that each HyD could detect spectral regions spanning from a 10-nm bandwidth up to the overall spectral capacity of the system (400–800 nm). Using this unique option, spectral scanning was per- formed on all the dyes to confirm signal specificity.

For 3D image data set acquisition, the excitation beam was first focused at the maximum signal intensity focal position within the sample and the appropriate HyD gain level was then selected to obtain the pixel intensities within range of 0–255 (8-bit images) using a color gradient func- tion. Later on, the beginning and end of the 3D stack (i.e. the top and the bottom optical sec- tions) were set based on the signal level degradation. Series of 2D Images for a selected 3D stack volume were then acquired with 1024X1024 pixels and were line averaged 3–4 times depending on the noise level. The 3D stack images with optical section thickness (z-axis) of approximately 0.3 micrometers were captured from cell volumes. For each cell volume reported here, z- section images were compiled and finally the 3-dimensional image restoration was performed using Imaris software (Bitplane).

### Preparation of MTF cultures for TEM

Cells were grown in culture as described, and then transferred to coverslips (Thermanox cover- slips, 15 mm D, Cat#72275–01) and grown for 3 additional days. Cells were then washed with ice cold 0.1 M sodium cacodylate (NaCAC)buffer, pH 7.3 three times for 5 min. Cells were fixed for 1 h at 4 C in 0.5% glutaraldehyde and 4% paraformaldehyde buffered with 0.1 M NaCAC buffer. They were again rinsed 3X with 0.1 M NaCAC at 4 C, and then post-fixed in 1% osmium tetroxide/1.5% potassium ferrocyanide overnight at 4 C in a wrapped container. Preparations were then rinsed with buffer, dehydrated in a graded series of ethanol, and embedded in Embed 812 (Electron Microscopy Sciences). A diamond knife mounted in a Porter-Blum MT-2B ultramicrotome was used to cut 70–90 nm thin sections. Sections were mounted on 200-mesh copper grids and stained with 2% aqueous uranyl acetate + lead citrate. Sections were examined in a Joel Jem 1400 TEM. An Orius SC1000 bottom-mounted CCD camera was used to capture the images.

### Single cell RNASeq analyses

We utilized microfluidic single cell capture and single cell mRNA sequencing technologies to explore genome-wide gene expression in PDAC cells. Fluidigm’s C1^TM^ Single-Cell Autoprep System (C1) allows fully automated capture of up to 96 single cells and subsequent cDNA synthesis for the use in QPCR and RNA-sequencing. Initial studies used C1 machines at the University of Pennsylvania and Yale, although our Sequencing Core facility has recently purchased a C1 machine which is currently being used. The cultured MTF cell suspension were loaded into the C1 and by using an integrated fluidic circuit chip, single cells were captured at distinct sites in microfluidic channels. Some heterogeneity in captured cells was evident in the capture sites. After optical confirmation of single cells at each capture site on the chip, the cells were processed for in-line cell lysis, reverse transcription, and cDNA amplification steps. The resultant cDNA was then subjected to Fluidigm’s BioMark DELTAgene QPCR assay using a custom-designed 36-marker PDAC panel for this purpose, which included EMT, macrophage, MTF, epithelial, and pancreatic markers (and housekeeping genes). We obtained single cell preparations from 5 patients (and have captured up to 90 single cells, with validated amplifications). Following validation of the QPCR marker panel, we then further utilized the amplified cDNA from each single cell to generate sequencing-ready libraries using Illumina’s Nextera XT library preparation kit. The libraries were pooled and sequenced by 1X50bp of total 150 million reads on an Illumina HiSeq 2500, which is sufficient in conducting comprehensive gene expression analysis.

#### Preprocessing of Illumina reads and transcriptome assembly

The quality of sequence read data for all single cell Illumina libraries was assessed using FastQC (http://www.bioinformatics.babraham.ac.uk/projects/fastqc/; version 0.11.5). Reads were trimmed using Trimmomatic v0.35 [118] with default settings at "ILLUMINACLIP:TruSeq3-SE:2:30:10 LEADING:3 TRAILING:3 SLIDINGWINDOW:4:15 MINLEN:36" to remove Illumina adapter sequences identified by FastQC and low-quality bases. Genome-guided (Ensembl build GRCh38.p7, http://www.ensembl.org/) and de novo assemblies of trimmed reads for all libraries were performed using Trinity r20140717 [119] and TopHat v2.0.14-Cufflinks v2.2.1 [120] respectively. The resulting transcriptome assemblies were each assessed for redundancy using GenomeTools v1.5.4 [121] to remove similar (sub)-sequences, then merged using CD-HIT-EST v4.6 [122]. Only the longest transcripts out of CD-HIT-EST clusters of sequences that shared at least 95% sequence similarity were retained for subsequent downstream analyses.

#### Expression quantification and quality control

Trimmed reads from each single cell library were mapped against the transcriptome assembly generated from all libraries using the Bowtie2 aligner [123]. The resulting alignment files (BAM files) were then used to estimate the transcript-level abundance by using the RSEM (RNA-Seq by Expectation Maximization) software [124]. TPM (Transcripts Per Million) unit was used as normalized expression values for each library. Expression matrices were created for sample libraries of each patient and combined sample libraries of all patients. Quality control was then performed on the expression matrices to identify and remove low-quality cells with a mapping rate of less than 50% of sequenced reads [125] and/or half a million total sequenced reads [125–128]. Additionally, transcripts for which more than 25% of cells showed zero expression across all cells [126] and those identified by the functional assignment as either mitochondrially encoded genes (mtDNA) or mitochondrially localized proteins [126, 129] were removed. Suitable samples from 4 patients were included.

#### Functional assignment

The assembled sequences were compared against 214,837 human cDNAs/non-coding RNA (ncRNA) annotated transcripts from Ensembl build GRCh38.p7 (blastn E-value = 1e-10) and 42,130 human proteins from UniProtKB/SwissProt (blastx E-value = 1e-5). Best scoring blast search targets when were then used to retrieve matching gene description and symbols from both Ensembl Biomart and UniProtKB online databases. KEGG pathway mapping and enrichment analysis of the expressed gene sets in the filtered expression matrices were performed using WebGestalt (WEB-based Gene SeT AnaLysis Toolkit) with the Ensembl human transcripts as reference background [130]. The enrichment analysis was performed using hypergeometric test and p-value (< 0.1) corrected by a Bonferroni multiple testing correction. Pathways selected for enrichment analysis were required to have a minimum of two genes.

#### Expression clustering

Heatmaps of filtered gene expression matrices were generated with Pheatmap R package version 1.0.8 [131]. Hierarchical clustering analysis was performed based on Euclidean distance to cluster rows (genes), and the cutree algorithm was used to divide rows into gene clusters with similar expression across single cells.

## Supporting information

S1 Spreadsheet Lists of genes contained within Clusters 1, 2, and 3. (XLSX)
